# EXPLORING SECTORAL REACH IN AGE-FRIENDLY COMMUNITIES

**DOI:** 10.1093/geroni/igad104.1507

**Published:** 2023-12-21

**Authors:** Kathy Black, Patricia Oh

**Affiliations:** University of South Florida,Sarasota-Manatee, Sarasota, Florida, United States; UMaine Center on Aging, Bangor, Maine, United States

## Abstract

There is increasing interest in better understanding the sectoral reach of age-friendly community practice. Action across a wide variety of actors is central to achieving age-friendly societal change according to the World Health Organization and an explicit goal for governmental participation in its Global Network of Age-Friendly Cities and Communities. However, there is limited knowledge regarding the scope of sectoral reach by age-friendly communities. We used qualitative inquiry to assess sectoral efforts reported by American age-friendly communities that completed a five-year cycle of participation (n=40). We employed directed content analysis using a priori indicators by sectoral actors: public (i.e., government), private (i.e., business), and civil society including nonprofit organizations and volunteers. We classify sectoral actions by type (i.e., intersectoral, multisectoral) and by clustered domain community foci (i.e., built, social, service). Our study identifies the extent and types of sectoral actors and actions reported by age-friendly communities with the greatest efforts reported in the public sector, and while similarly distributed across all the domains, slightly more efforts were noted in the built environment. We also found greater intersectoral efforts (i.e., explicitly working towards shared goals) than multisectoral (i.e., not necessarily in collaboration on shared goals), particularly across government. While our study substantiates the breadth of actions towards age-friendly change, additional research is needed to examine the ways in which the public and other sectoral actions are further linked to outcomes in communities in the United States and in other countries.

